# *In vivo* effects of temperature on the heart and pyloric rhythms in the crab *Cancer borealis*

**DOI:** 10.1242/jeb.199190

**Published:** 2019-03-01

**Authors:** Dahlia Kushinsky, Ekaterina O. Morozova, Eve Marder

**Affiliations:** Biology Department and Volen Center, Brandeis University, Waltham, MA 02454, USA

**Keywords:** Photoplethysmography, Crustaceans, Stomatogastric nervous system, Cardiac ganglion, Central pattern generator, *Q*_10_

## Abstract

The heart and pyloric rhythms of crustaceans have been studied separately and extensively over many years. Local and hormonal neuromodulation and sensory inputs into these central pattern generator circuits play a significant role in an animal's response to perturbations, but are usually lost or removed during *in vitro* studies. To examine simultaneously the *in vivo* motor output of the crustacean heart and pyloric rhythms, we used photoplethysmography. In the population measured (*n*=49), the heart rhythm frequency ranged from 0.3 to 2.3 Hz. The pyloric rhythm varied from 0.2 to 1.6 Hz. We observed a weak correlation between the frequencies of the heart and pyloric rhythms. During multiple hour-long recordings, many animals held at a controlled temperature showed strong inhibitory bouts in which the heart decreased in frequency or become quiescent and the pyloric rhythm decreased in frequency. We measured the simultaneous responses of the rhythms to temperature ramps by heating or cooling the saline bath while recording both the heart and pyloric muscle movements. *Q*_10_, critical temperature (temperature at which muscle function is compromised) and changes in frequency were calculated for each of the rhythms tested. The heart rhythm was more robust to high temperature than the pyloric rhythm.

## Abstract (precis)

**Summary:** Temperature elevation increases the frequency of the heart and pyloric rhythms of the crab *Cancer borealis*, but the heart rhythm has a higher critical temperature than the pyloric rhythm.

## INTRODUCTION

Central pattern generators (CPGs) are neuronal circuits that produce rhythmic motor patterns. In crustaceans, mechanisms of central pattern generation have been studied using the cardiac ganglion (CG), which produces heart movements, and the stomatogastric ganglion (STG), which generates the rhythmic movements of the stomach (Cooke, 2002; Maynard, 1972). These ganglia, when isolated, can generate rhythmic motor patterns *in vitro* that resemble those seen *in vivo.* The heart is found in the animal just posterior to the stomach, and pumps hemolymph through the ophthalmic artery, directly bathing the STG. Although there are no known direct neuronal connections between these ganglia, we were curious to know whether these two rhythms are coordinated in the intact animal.

The crustacean stomach is a complex mechanical structure with ossicles that provide mechanical support and insertions for intrinsic stomach muscles (Maynard and Dando, 1974). Driving the movements of the stomach, the STG is composed of approximately 26–30 identified neurons, which generate the continuously active and rapid (∼1 Hz) pyloric rhythm and the slower episodic gastric mill movements (Clemens et al., 1998a,b,c, 1999, 2001; Heinzel, 1988; Heinzel and Selverston, 1988; Rezer and Moulins, 1983; Soofi et al., 2014). The heart of the crab, *Cancer borealis*, is driven by the CG, which is composed of nine neurons, four small and five large cells, that burst in time to produce muscle movements of the single chambered heart. In addition to the CG pacemaker neurons, the heart is innervated by extrinsic fibers, one inhibitory and two excitatory, that modify heart rate in relation to behavioral demands. The cardiac rhythm frequency is highly variable but lies within the same range as the pyloric frequency.

Neurohormones are delivered throughout the animal via the hemolymph (Alexandrowicz and Carlisle, 1953; Chen et al., 2010; Christie et al., 1995; DeKeyser et al., 2007; Hui et al., 2012). Under normal conditions, the heart continuously pumps hemolymph through an open circulatory system to distribute oxygen, nutrients and neuromodulators (McMahon, 1995; McMahon, 2001a,b). Hemolymph properties, such as oxygen tension and neuromodulator content, will alter the neuronal activity of the STG and therefore its motor output (Clemens et al., 2001). Neurohormones may increase the frequency and amplitude of the heartbeat (Christie et al., 2008; Cruz-Bermudez and Marder, 2007; Sullivan and Miller, 1984; Williams et al., 2013), or change the frequency of the pyloric rhythm (Hooper and Marder, 1987; Marder, 2012; Marder and Bucher, 2007). Any given modulator may affect numerous organs in a coordinated manner, or may have varied effects depending on the receptors present and the state of the target tissue. This provides a potential feedback system within the animal, allowing for the correlated adjustment of organ activity in the animal. Thus, one of the questions addressed in this study is whether changes in heart activity, for example periods of bradycardia (McMahon, 1999) during which the heart slows or completely stops, are associated with movements of the stomach.

The temperature of the animal's environment also plays a drastic role in altering activity of the STG and CG. Crustaceans are poikilotherms and therefore may experience wide variations in body temperature due to environmental temperature changes. These can be both short-term temperature fluctuations, due to local environmental temperature change through changing tidal patterns during a single day, and long-term temperature fluctuations, due to seasonal temperature variations (Marder et al., 2015; Soofi et al., 2014; Tang et al., 2010, 2012).

Previous work in crustaceans indicates that both the heart and pyloric rhythms are robust to temperature changes. Studies in the heart have shown that the strength of a heartbeat (directly related to stroke volume) decreases and heart rate increases with increases in temperature (Camacho et al., 2006; Worden et al., 2006). Therefore, the increase in heart rate partially compensates for the decrease in stroke volume as the CG is further pushed from its normal state. The triphasic pyloric rhythm is maintained across a wide range of temperatures. In both heart and pyloric rhythms, the maximum frequency attained at the highest temperatures *in vivo* is lower than those of *in vitro* conditions, likely because of the presence of sensory feedback and neurohormonal input (Soofi et al., 2014; Worden et al., 2006).

Temperature increases beyond 23°C can lead to severely disrupted motor patterns and subsequent loss of stereotypical rhythmic muscle movements in the heart and pyloric rhythms (Haddad and Marder, 2018; Rinberg et al., 2013; Marder et al., 2015; Tang et al., 2012). Recordings of the pyloric rhythm indicate that preparations behave similarly at lower, permissive temperatures, but extreme temperatures disrupt, or crash, preparations in dissimilar ways, revealing their variability (Haddad and Marder, 2018; Tang et al., 2012).

In this study, we systematically explored the potential relationships between stomach and heart rhythms, and asked whether they are coordinately sensitive to perturbation by temperature.

## MATERIALS AND METHODS

### Animals

Adult male Jonah crabs (*Cancer borealis* Stimpson 1859) between 400 and 700 g were obtained from Commercial Lobster (Boston, MA, USA). Animals were housed in tanks with artificial seawater (Instant Ocean) held between 10 and 13°C on a 12 h light:12 h dark cycle without food for a maximum of 10 days. Experiments were done between 1 September 2016 and 12 July 2017.

**Fig. 1.**
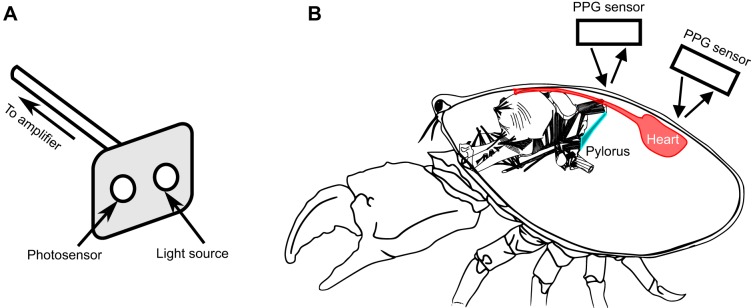
**Use of photoplethysmography (PPG) allows for continuous non-invasive recording of the heart and pyloric muscles.** (A) Drawing of the PPG sensor system, indicating the light source and photosensor of the device. (B) Drawing of the PPG sensor positioning for detection of heart and pylorus movements when placed on the carapace of an animal. Infrared light from the sensors is emitted and travels through the animal's carapace before being reflected from the muscle directly below the PPG sensor. Crab image modified from http://stg.rutgers.edu/Resources.html. Stomach image modified from Maynard and Dando (1974).

Prior to each experiment, crabs were weighed and anesthetized on ice for 10 min. Photoplethysmogram (PPG) sensors (Vishay CNY70331, emitter wavelength 950 nm), as described in Depledge (1983), were placed on the carapace above the heart and pyloric muscles to record the heart and pyloric rhythms, respectively (Fig. 1). Sensors were secured to the carapace using dental wax and cyanoacrylate glue (Starbond, EM-2000) and covered in Marine Adhesive Sealant (3M, Fast Cure 5200) to waterproof them and ensure their stability. After the sensors had been attached to the animal, we waited a minimum of 16 h prior to experimental recordings, during which time the animals were not handled and the door to the incubator was not opened.

### Temperature experiments

After at least 30 min of baseline recording (10–12°C) the water temperature was changed by flowing either cold or warm saline into the tank through a tube inserted through the door of the incubator. A vacuum line was used to pump water out of the tank to maintain a constant volume of saline. The temperature was slowly ramped from 11°C to 32°C over 1.5–2 h. The heart rate was closely monitored to ensure the animal's health during the temperature changes and the ramps were halted once the heart rate developed an arrhythmia or decreased to frequencies similar to those at a baseline temperature of 11°C, indicating that a ‘critical temperature’ had been reached. Increasing temperature past this critical temperature led to the death of the animal, as past this temperature the heart no longer recovered functionality.

### Data acquisition and analysis

PPG data were acquired through the PPG amplifier (Newshift AMP03) and recorded digitally through a digitizer (Axon Digidata 1550B) into computer software (AxoScope 10.6) with a sampling frequency of 500 Hz. Data were analyzed using custom-written C and Matlab codes.

### Electromyographic recordings

Electrodes made out of 0.38 mm Teflon-coated stainless steel wire were inserted into the crab though small holes (1–2 mm diameter) made in the carapace. Recordings were amplified using an A-M Systems differential amplifier. Data were digitized using a DigiData 1440A (Axon Instruments) and recorded using pClamp software (Axon Instruments). Electrodes were positioned and held with micromanipulators, and inserted through the holes in the general location of the heart and pyloric muscles until successful implants and signal-to noise ratio were established. Crabs were kept on ice (at ∼4°C) during the electromyographic (EMG) recordings.

### Analysis of heart rhythm frequency

The heart rhythm frequency was calculated as the frequency at the peak spectral power. We used the Burg (1967) method to estimate the power spectral density at each moving-time window. The Burg (1967) method fits the autoregressive model of a specified order *P* in the time series by minimizing the sum of squares of the residuals. The fast-Fourier transform (FFT) spectrum was estimated using the previously calculated autoregressive coefficients. This method is characterized by higher resolution in the frequency domain than traditional FFT spectral analysis, especially for a relatively short time window (Buttkus, 2000). We used the following parameters for the spectral estimation: data window of 12.8 s (128 samples), 50% overlap to calculate spectrogram, number of estimated autoregressive coefficients *P*=window/4+1. Before the analysis, the voltage offsets of the PPG recordings were removed, low-pass filtered to 5 Hz using a six-order Butterworth filter and downsampled. Average baseline frequencies of heart rhythm were calculated as median frequencies at the peak of the power spectral density for each window of a spectrogram during baseline conditions. Cumulative histograms of baseline frequencies were calculated as a sum of histograms from individual animals normalized so that the sum of bar heights is less than or equal to 1.

### Analysis of the pyloric rhythm frequency

The pyloric rhythm frequency was calculated in a similar way to heart frequency in those cases that showed no interference from heart activity. However, in many instances the spectrogram of the pyloric recording showed power at two frequencies, one at the pyloric rhythm frequency and the other at the heart rhythm frequency. In these cases, to identify the pyloric rhythm frequency, a linear regression model was fitted to the pyloric signal while taking into account the phase difference between the heart and pyloric signals. The heart signal was multiplied by the coefficients of the regression model and subtracted from the pyloric signal. Then, the spectrogram of the subtracted signal was calculated, and the pyloric frequency was identified as the frequency at the peak spectral power. In rare cases, the pyloric rhythm frequency could not be determined because of irregularities in the signal.

### Analysis of the inhibitory bouts

We used a hidden Markov model (HMM) to infer the active and inhibitory states of the heart rhythms. In HMM, a time series is modeled as being generated probabilistically from an underlying discrete-valued stochastic process (Rabiner, 1989). The data can be either discrete valued or continuous valued, while the unobservable ‘hidden’ state is a discrete random variable that can take *n* possible values (in our case *n*=2, representing active and inhibitory states). Estimation of the transition probabilities for HMM was done using the Baum–Welch algorithm, which utilizes an expectation maximization (EM) algorithm (Bilmes, 1998). The initial parameters used for the detection were as follows: transition matrix *P*_AI_=*P*_IA_=0.9, *P*_AA_=*P*_II_=0.1, where *P*_AI_ is the transition probability from active to inhibitory state, *P*_AI_ is the transition probability from inhibitory to active state, *P*_II_ is the transition probability from inhibitory to inhibitory state, and *P*_AA_ is the transition probability from active to active state. Identification of HMM states was used to calculate the durations of active and inhibitory bouts of the heart rhythms.

### Coherence analysis

The coherence between heart PPG and pyloric PPG was calculated with multi-taper Fourier analysis (Mitra and Pesaran, 1999) using the Chronux toolbox (http://chronux.org). Data were binned into 2 min bins, moved in 5 s steps. The time–bandwidth product was set to 10, and 19 tapers were used. The peak coherence and its frequency were calculated for each window. The theoretical confidence level of the coherence (*C*) was calculated as follows:
(1)
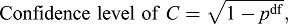
where

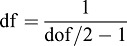
and dof is degrees of freedom. Based on these calculations, coherence values >0.625 reached significance at *P*<0.05 with a Bonferroni correction. Datasets that had significant coherence more than 50% of the time during the baseline were considered to have significantly coherent heart and pyloric recordings and were calculated for each dataset. The phase difference between the heart and pyloric recordings was calculated at the frequency of the peak coherence for each window and the median value of the phase difference was reported for each dataset with significantly coherent signals.

### *Q*_10_ estimation

We estimated the *Q*_10_ of the heart and pyloric rhythms *in vivo*. The frequency (*F*) of the heart and pyloric rhythms was plotted as a function of temperature (*T*) on a logarithmic scale, and the *Q*_10_ was extracted from the slope of the linear regression (*m*) following the equation:
(2)
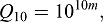
where

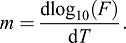
The goodness of fit of the linear regression model for each dataset was assessed by calculating the coefficient of determination *R*^2^, calculated as *R*^2^=(correlation coefficient)^2^. We report *R*^2^ for heart and pyloric data in Table 1. For the majority of the fits, we obtained high values of *R*^2^>0.8.

**Table 1.**
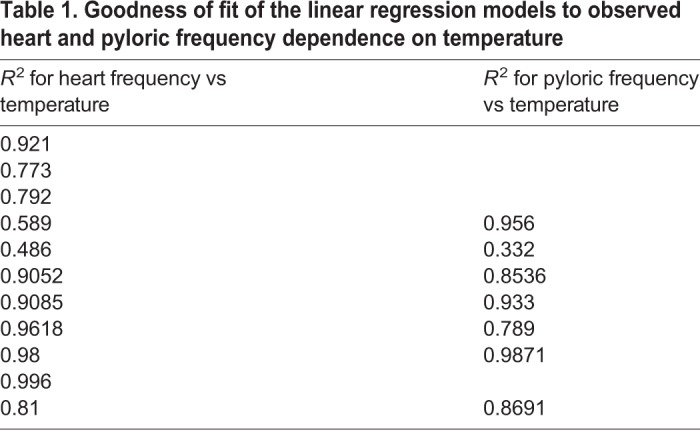
**Goodness of fit of the linear regression models to observed heart and pyloric frequency dependence on temperature**

### Critical temperature analysis

The critical temperature was defined as the temperature at which the heart and pyloric recordings became irregular and the frequency decreased. This was determined from the spectrograms of the heart and pyloric rhythms. In some cases, the critical temperature of the pyloric rhythm was impossible to determine as a result of irregularities in the pyloric rhythm signal.

### Statistical analysis

All statistical analyses were done in Matlab. Between-group comparisons were done using one-way ANOVA. The significance level was set to 0.05.

## RESULTS

Heart and pyloric muscle movements of *C. borealis* were recorded *in vivo* using PPG (Fig. 1). In the majority of experiments, both rhythms were recorded from the same animal. Some experiments included recordings of an animal's heart rhythm only. The placement of the PPG sensor over the heart is straightforward, as the heart is dorsal, and situated just under the carapace, with large movements almost in the same plane as the sensor itself. The ophthalmic artery exits the heart and travels anteriorly close to the surface of the stomach. The pylorus is the most posterior region of the stomach, but the pylorus moves orthogonally to the carapace, making it more difficult to capture its movements from the surface of the animal. The thin straps of the dorsal dilator muscles of the pyloric region connect the pylorus to insertions just below the carapace, and it is the movements of these muscles that the successful PPG recordings were picking up. Additionally, each heart beat elicits a pressure wave in the artery. As the artery runs between the bilaterally symmetrical dorsal dilator muscles, it is not surprising that the pyloric PPG sensor might pick up traces of the heart rhythm, most likely through pulsations of the ophthalmic artery.

**Fig. 2.**
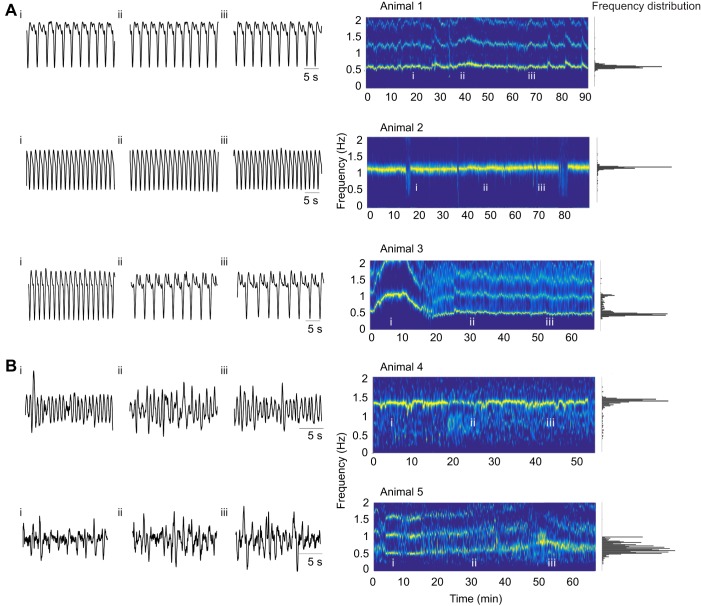
**Variability of heart and pyloric rhythm frequency under baseline conditions.** (A) Heart rhythm recordings from three animals at different times under baseline conditions (left) with their spectrograms (right). Roman numerals mark where 30 s raw traces were obtained in the longer baseline frequency data (spectrograms). (B) Pyloric recordings from two animals under baseline conditions (left) with their spectrograms (right).

Fig. 2 shows raw data and associated spectrograms from heart recordings from three individual animals (Fig. 2A) and from pyloric recordings from two individual animals (Fig. 2B). We include these here to allow the reader to appreciate the kinds of animal-to-animal variability seen in the raw data. Animal 1 had a relatively slow and steady heart rhythm at 0.6 Hz, while animal 2 had a relatively high frequency (1.1 Hz) and steady heart rhythm. The regularity of these rhythms is seen in the single peaks in their frequency distributions. Animals 1 and 2 were typical of the majority of animals, and were relatively stationary during their baseline recordings. In contrast, animal 3 showed spontaneous frequency changes under control conditions. We include this here to illustrate that some individual animals showed non-stationary behavior, and this was associated with bimodal or otherwise complex frequency distributions.

**Fig. 3.**
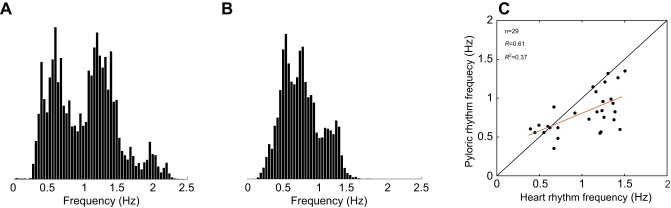
**Frequency distributions of the heart and pyloric rhythms.** (A) The sum of all of the normalized frequency distributions of the heart rhythms from 49 animals under baseline conditions. (B) The sum of the normalized frequency distributions of the pyloric rhythms from 29 animals under baseline conditions. (C) Plot of the baseline heart rhythm frequency versus pyloric rhythm frequency for those animals for which both rhythms were recorded. The red line is the linear regression of the data (*R*=0.61 and *R*^2^=0.37).

PPG recordings placed over the pylorus yielded complex waveforms that were more variable than those of the heart (Fig. 2B). The variability of the actual waveform is largely attributable to the angle of the dorsal dilator muscle movement with respect to the PPG sensor placement on the carapace of the animal. The frequency of the pyloric rhythm recorded this way is within the range of those recorded *in vitro* or with electrodes *in vivo* (Hamood and Marder, 2015; Soofi et al., 2014). Animal 4 maintained a stable pyloric rhythm frequency, while animal 5 showed a more variable rhythm during its baseline period. Frequency data from the PPG recordings of the heart (49 animals) and the pylorus (29 animals) were calculated (Fig. 3). In all cases, the data came from stretches of recordings in excess of 30 min. All pyloric rhythm data are from animals that were also used for heart measurements. The histogram in Fig. 3A shows an apparent multimodal distribution of heart frequencies (Hartigan's dip test, dip=0.067, *P*=0.029). Heart rhythm frequencies ranged between 0.4 and 2.4 Hz (Fig. 3A). No correlation was found between heart rhythm frequency and time of year of experiment (*P*=0.25, Pearson's correlation coefficient). Additionally, no correlation was found between average heart rhythm frequency and crab mass (*P*=0.37, Pearson's correlation coefficient).

The distribution of the pyloric rhythm frequencies was close to normal, typical of other reports of pyloric rhythms (Fig. 3B). The spread in pyloric rhythm frequencies was much smaller than that in heart frequencies, ranging from 0.2 to 1.6 Hz. The frequency of the pyloric rhythm was plotted as a function of the heart rhythm for the 29 animals for which we had measurements of both the heart and pyloric rhythms (Fig. 3C). More than half of the points were dispersed away from the identity line, demonstrating that while the pyloric and heart frequencies are in the same general range, within an animal they are not identical. Nonetheless, there was a modest correlation (*R*=0.61) between the frequencies of the two rhythms (Fig. 3C).

### Inhibitory bouts

**Fig. 4.**
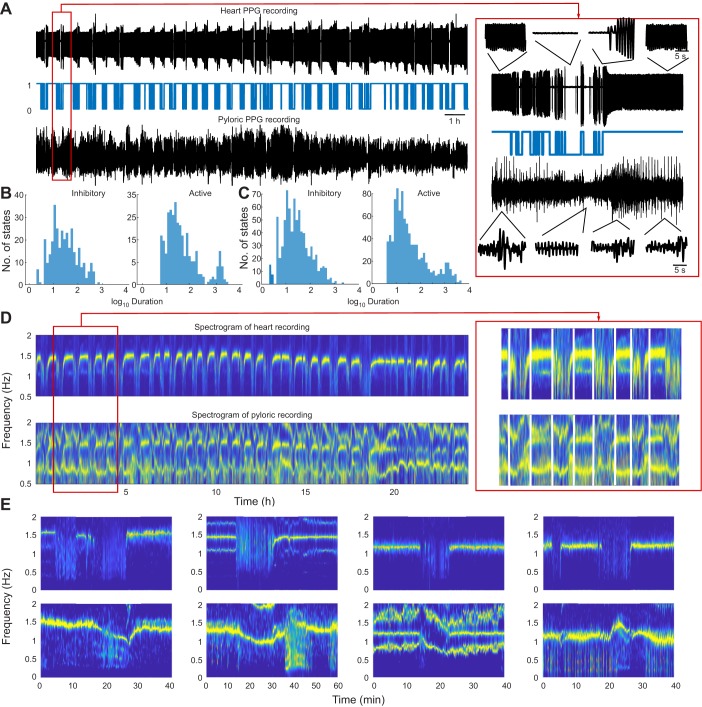
**The heart rhythm exhibits inhibitory bouts that influence the pyloric rhythm.** (A) Top panel: a 24 h recording of the heart rhythm with inhibitory bouts throughout the recording session. Middle panel: states identified from the heart rhythm trace using a hidden Markov model. State 1 corresponds to an active state and state 0 corresponds to an inhibitory bout. Bottom panel: simultaneously recorded pyloric rhythm. Expanded traces during a single inhibitory bout (red box) are shown on the right. (B) Distribution of durations (in s) of active and inhibitory states of the heart rhythm shown in A, identified using the hidden Markov model, using baseline data. (C) Cumulative distribution of the durations (in s) of active and inhibitory states of the heart activity from 20 animals that showed inhibitory bouts with a mean duration of 30 s. Active state durations displayed a bimodal distribution. (D) Top panel, spectrogram of the heart rhythm shown in A. The heart rhythm frequency between the inhibitory bouts was relatively constant at 1.5 Hz. Bottom panel, spectrogram of the pyloric rhythm shown in A. The pyloric rhythm decreased in frequency during the heart inhibitory bouts followed by an increase in frequency at the end of the bouts. An enlarged portion of the spectrogram (red box) is shown on the right. (E) Additional examples of the frequency changes of the pyloric rhythm during and following the heart inhibitory bouts from four different animals. Top panels, spectrograms of heart activity. Bottom panels, spectrograms of pyloric activity. These data feature an interaction between the cardiac and the pyloric activity on the time scale of minutes.

The heart rhythms often displayed periods of bradycardia, during which the heart considerably slowed or halted for a significant period of time (Fig. 4). We identified these inhibitory bouts using a hidden Markov model (see Materials and Methods). A 24 h recording with multiple inhibitory bouts is shown in Fig. 4A. The termination of the inhibitory bouts was associated with a recovery of both the amplitude and frequency. A temporary increase in the heart signal amplitude was sometimes observed immediately following an inhibitory bout (Fig. 4A, right). Inhibitory bouts were seen in 41% (20/49) of the tested population. When inhibitory bouts were seen, they often occurred repeatedly over extended periods of time, such as those in the 24 h recordings shown in Fig. 4. Inhibitory bout duration and frequency were variable both across and between animals (Fig. 4B), and were not significantly correlated with the time of year (*P*=0.79, Pearson's correlation coefficient) or animal mass (*P*=0.41, Pearson's correlation coefficient).

Long periods of bradycardia affected the frequency and amplitude of the pyloric rhythm. Simultaneously recorded pyloric and heart signals in long-term experiments showed that the amplitude of the pyloric signal decreased during the heart inhibitory bouts (Fig. 4A). The durations of the inhibitory states were very variable (Fig. 4B,C), while the distributions of the durations of the active states were also variable, but bimodal (Fig. 4B,C).

Only 5 of the 20 animals that exhibited periods of bradycardia had long inhibitory bouts of the order of tens of minutes. During these bouts, changes in pyloric frequency were detected (Fig. 4D,E). There was considerable variability in the frequency changes of the pyloric rhythm, as can be seen from the spectrograms calculated during a single inhibitory bout in four animals (Fig. 4E). The first panel of Fig. 4E (from left to right) illustrates an example of when the pyloric rhythm was reliably active at 1.5 Hz while the heart was beating at 1.6 Hz. In this animal, when the heart temporarily stopped, the pyloric rhythm slowed to 0.9 Hz. The second panel also shows a strong decrease in pyloric frequency during the heart inhibitory bout. The third panel shows a transient increase followed by a decrease, while the fourth panel shows a slight increase in the pyloric rhythm frequency.

### Relationships between the heart and pyloric rhythms

Although it is clear that the pyloric and heart rhythms are often at different frequencies, and the pyloric rhythm continues during the inhibitory bouts, spectrograms of the pyloric PPG recordings often reveal a band at the heart frequency. In one example shown in Fig. 4D, the heart rhythm had a tight frequency band around 1.2 Hz. That same band was also seen in the pyloric PPG spectrogram. When the heart stopped, the heart band disappeared from both recordings.

To determine the potential influence of the heart rhythm on the pyloric recording, we calculated the time–frequency coherence between simultaneously recorded heart and pyloric PPG signals in 2 min bins moved in 5 s steps. In 70% of the animals (28/40), the heart and pyloric signals were significantly coherent at the frequency of the heart more than 50% of the time during the baseline period. Because the pyloric frequency often changed when the heart stopped, this suggests a possible biomechanical coupling or common drive influencing the two structures.

**Fig. 5.**
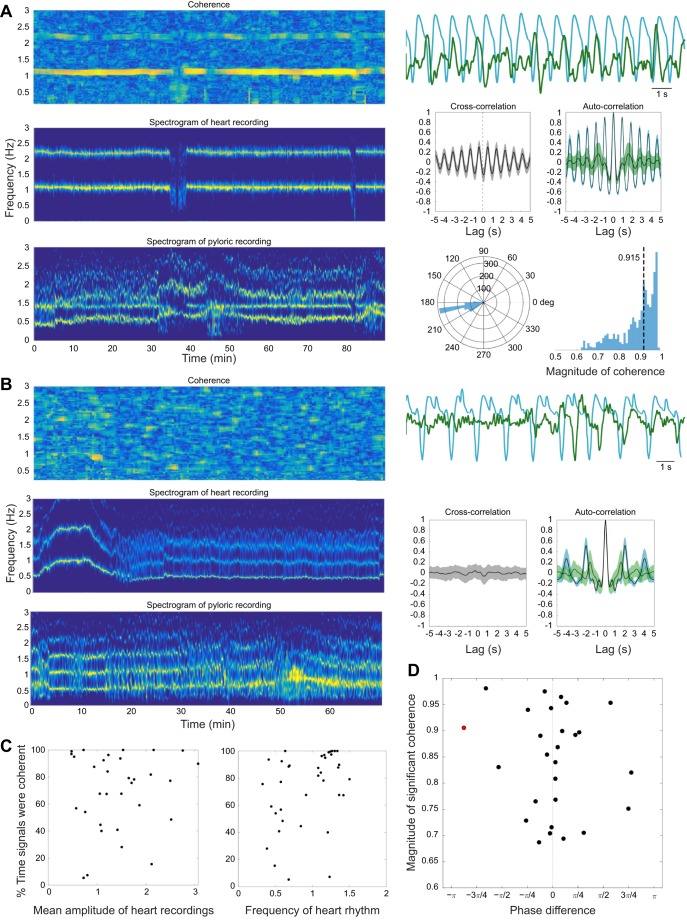
**Pyloric and heart recordings often show coherence at the frequency of the heart rhythm.** (A) An example of high coherence between heart and pyloric PPG recordings. Left, top panel: time–frequency coherence of the heart and pyloric recordings at baseline. Left, middle and bottom panels: spectrograms of the heart and pyloric recordings at baseline. Right, top panel: 15 s segments of heart rhythm (blue) and pyloric recordings (green), simultaneously recorded under baseline conditions. Right, middle panel: heart–pyloric cross-correlation (gray) and autocorrelation functions of heart (blue) and pyloric (green) recordings. Right, bottom panel: the distribution of phase differences between the heart and pyloric recordings calculated in 2 min windows, moved in 10 s steps (left) and the distribution of magnitudes of peak coherence calculated in 2 min windows, moved in 10 s steps (right). (B) Example of the absence of coherence between the heart and pyloric rhythms. Left, top panel: time–frequency coherence of heart and pyloric rhythms at baseline. Left, middle and bottom panels: spectrograms of heart and pyloric rhythms at baseline. Right, top panel: heart rhythm (blue) with simultaneously recorded pyloric rhythm (green) recorded at baseline for 30 s segments of the full data range for which spectra and coherence were calculated. Right, bottom panel: heart–pyloric cross-correlation (gray) and autocorrelation functions of heart (blue) and pyloric (green) rhythms. (C) Coherence statistics for all animals with simultaneous recordings of heart and pyloric PPG recordings (*n*=40). Left: percentage of time pyloric and heart recordings were significantly coherent at baseline versus the amplitude of the recorded heart signal. Right: percentage of time pyloric and heart recordings were significantly coherent at baseline versus heart rhythm frequency. (D) Scatter plot showing median magnitudes of peak coherence versus median phase difference between heart and pyloric recordings.

In the experiment shown in Fig. 5, the coherence peaked at 1.1 Hz (Fig. 5A), which was the heart frequency. The spectrogram of the pyloric recording has two frequency bands: one at approximately 0.5 Hz, which is the intrinsic frequency of the pyloric oscillations, and the other at the heart rhythm frequency. By calculating the phase difference between the heart and pyloric rhythms at the frequency of the peak coherence, we determined that the pyloric rhythm was shifted by 190 deg relative to the heart rhythm. This means that, in this animal, the heart and pyloric rhythms were almost in antiphase. This can also be seen from the cross-correlation function, which has a minimum close to zero lag. We also calculated auto-correlations for the heart and pyloric rhythms. The pyloric rhythm has a more complex auto-correlation function than the heart rhythm and features two peaks, one at the lag corresponding to the period of the heart oscillation, and the second at the period of intrinsic pyloric oscillations. However, not all animals showed coherence between the heart and pyloric recording (Fig. 5B). Overall, 28/40 animals had significant coherence between the recordings. Thus, in many animals the heart rhythm appears as a second frequency band in the pyloric PPG spectrogram. There was no dependence of the percentage of time the rhythms were significantly coherent on the amplitude or frequency of the heart signal (Fig. 5C). In many animals, the heart and pyloric signals were coherent at close to zero phase lag (Fig. 5D).

To determine whether the coherence between the two rhythms seen in the PPG recordings is also seen at the level of motor neuron discharge *in vivo*, we performed EMG recordings from the heart and the pyloric muscles (*n*=3) together with the PPG recordings of the heart. We did not observe any coherence between the heart and pyloric EMG recordings in any of these experiments (data not shown).

### Effects of temperature on the heart and pyloric rhythms

**Fig. 6.**
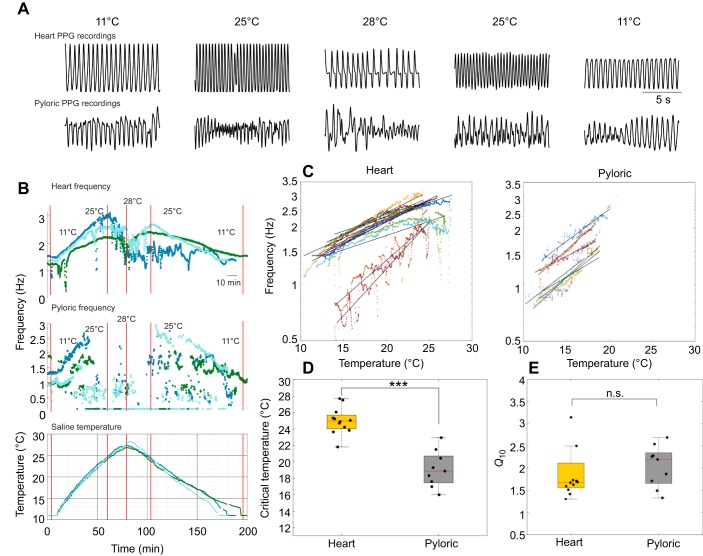
**The pyloric rhythm is more sensitive to increases in temperature than is the heart rhythm.** (A) Raw traces of heart (top) and pyloric (bottom) muscle activity at baseline (11°C), and during increasing (25°C), critical (28°C) and decreasing portions (25°C) of the temperature ramp. (B) Top panel: change in frequency of heart rhythms of three animals in response to almost identical temperature ramps shown in the bottom panel. Middle panel: change in frequency of simultaneously recorded pyloric rhythm in response to the temperature ramp. The pyloric rhythm is less robust to temperature increases than the heart rhythm and crashes at a much lower temperature. The crash is evident from a significant decrease in frequency and amplitude of the pyloric rhythm. (C) Frequencies of the heart and pyloric rhythms during portions of increasing temperature ramps plotted as a function of temperature on a logarithmic scale. Each color corresponds to an individual animal. A line was fitted to data points for each animal's heart frequencies to estimate the *Q*_10_. (D) Critical temperature of the heart rhythm is significantly higher than that of the pyloric rhythm (mean heart critical temperature 25.0±1.6°C, mean pyloric critical temperature 19.1±2.8°C, one-way ANOVA, ***P=0.0005, *F*_1,19_=21.07). (E) *Q*_10_ values of heart and pyloric frequencies are not significantly different (mean *Q*_10_ of heart frequency 2.007±0.854, pyloric frequency 2.040±0.467, one-way ANOVA, *P*=0.9155, *F*_1,19_=0.01).

We tested the effects of increasing temperature on the heart movements of 12 animals and on pyloric movements in nine animals (Fig. 6). The saline temperature was ramped from 11°C to 28°C while recording the heart and pyloric rhythms. As the temperature increased, the heart frequency increased until the heart rhythm became irregular and ‘crashed’. Crashes were reversible, as activity recovered when the temperature was decreased. The maximal frequencies, critical temperatures (temperature at which irregularities started to occur) and recovery patterns were different across animals (Fig. 6B). The frequency of the pyloric rhythm also increased with increasing temperature, again characterized by irregular periods of crashed activity at high temperatures. Additionally, the pyloric waveforms often changed in shape, exhibiting peaks followed by long plateaued waveforms.

The pyloric rhythm consistently crashed at lower temperatures than the heart rhythm in the same animal. The mean (±s.d.) heart critical temperature was 25.0±1.62°C and the mean pyloric critical temperature was 19.1±2.76°C (Fig. 6D). The critical temperatures of the heart and pyloric muscle movements were significantly different (one-way ANOVA, *P*=0.0005, *F*_1,19_=21.07).

The frequencies of the heart and pyloric rhythms are plotted as a function of temperature on a logarithmic scale in Fig. 6C. Linear models were fitted to the data for each animal to estimate the *Q*_10_ of the heart and pyloric frequencies (Fig. 6C). The *Q*_10_ of the heart rhythms ranged from 1.3 to 4.2 with a mean (±s.d.) of 2.0±0.85. Pyloric rhythm *Q*_10_ ranged from 1.3 to 2.7 with a mean (±s.d.) of 2.07±0.47. The *Q*_10_ values of the heart and pyloric rhythms were not significantly different as determined by one-way ANOVA (*P*=0.127, *F*_1,19_=2.54) (Fig. 6E). Coefficients of determination (*R*^2^) for each fit are shown in Table 1.

**Fig. 7.**
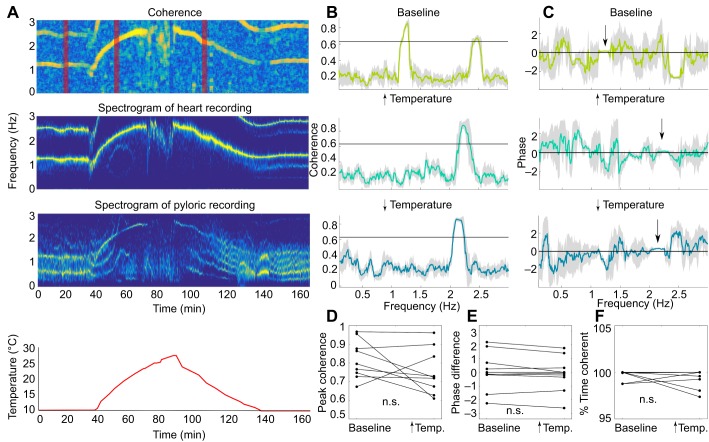
**Coherent heart and pyloric recordings remain coherent during the temperature ramp without change in phase relationship.** (A) An individual example of coherence between the heart and pyloric recordings and spectrograms of the recordings at baseline and during the temperature ramp. Bottom panel shows tank temperature. (B) Examples of coherence at baseline during temperature increases and temperature decreases. Horizontal black lines indicate the theoretical significance level. The times at which example coherences were taken are shown by the vertical red bars in A. Recordings are coherent throughout the whole experiment at the frequency of the heart rhythm. (C) Phase difference of the rhythms in A at baseline, and during rising and decaying phases of the temperature ramps. (D) Peak coherence between heart and pyloric recordings from all temperature experiments (*n=*9) at baseline and during the rising phase of the temperature ramp. Coherence during the temperature ramp was calculated up to the critical temperature of the pyloric rhythm. There were no statistically significant differences between mean peak coherence at baseline and during the temperature ramp as determined by one-way ANOVA (*P*=0.806, *F*_1,32_=0.06). (E) Phase difference between heart and pyloric recordings for all temperature experiments (*n=*9). There were no statistically significant differences between mean phase differences at baseline and during the temperature ramp as determined by one-way ANOVA (*P*=0.759, *F*_1,32_=0.1). (F) Percentage of time heart and pyloric recordings were coherent. There were no statistically significant differences between mean percentage of time the recordings were coherent at baseline and during the temperature ramp as determined by one-way ANOVA (*P*=0.354, *F*_1,16_=0.91). In all nine temperature experiments, recordings showed coherence more than 95% of the time.

We calculated the coherence between the heart and pyloric rhythms during the temperature ramps to determine whether temperature perturbation affected the relationship between the signals. An example of time–frequency coherence from an individual animal is shown in Fig. 7A. The coherence peaked at the frequency of the heart oscillations both at baseline temperatures and during the temperature ramps. Examples of coherence and phase differences calculated at baseline, during the rising phase of the temperature ramp, and during the decreasing phase of the temperature ramp show that the amplitude of the coherence remained high throughout the experiment and the temperature perturbation did not affect the phase relationship between the rhythms. In this example, the rhythms were in phase (phase difference at the frequency of maximal coherence is shown by the arrows in Fig. 7C). Data from nine experiments with simultaneously recorded heart and pyloric rhythms are summarized in Fig. 7D. The coherence was calculated up to the point of the pyloric rhythm crash. Neither mean peak coherence nor mean phase differed between the recordings taken at baseline and those taken during the temperature ramps (one-way ANOVA, *P*=0.806, *F*_1,32_=0.06; one-way ANOVA, *P*=0.759, *F*_1,32_=0.1). Finally, the recordings were coherent for approximately the same amount of time at baseline and during the rising phase of the temperature ramp (one-way ANOVA, *P*=0.354, *F*_1,16_=0.91). These data suggest that rhythms that were coherent at baseline remained coherent during the increase of temperature without a significant change in phase relationship. This is consistent with the interpretation that the coherence between the pyloric PPG and the heart PPG is largely attributable to the pyloric PPG picking both the pyloric-timed movements driven by the STG and a signal from the heart itself.

## DISCUSSION

It is informative to know when different body rhythms show correlated activity patterns. It has been previously shown that the crab heart rhythm is coordinately regulated with its ventilatory rhythms (McMahon, 1999), suggesting the possibility that other physiological processes in the animal might also be coordinately regulated. The crustacean pyloric and heart rhythms have similar frequencies, and the STG is situated just anterior to the heart in the ophthalmic artery, making it likely that the heart and the STG receive similar hormonal influences. We therefore asked whether these rhythms are likely to be coordinately regulated in the intact animal, especially because both the STG and heart respond to a vast array of the same modulatory influences (Christie et al., 2010; Cruz-Bermudez and Marder, 2007; Dickinson et al., 2016). To the best of our knowledge, ours is the first study to record simultaneously the heart and pyloric rhythms both under control conditions and in response to temperature changes. Although the frequencies of the rhythms were not the same, there was a modest correlation between them which could be attributable to some common neurohormonal influences.

### Baseline functions

We captured heart activity using non-invasive PPG recordings that limit the stress placed on the animal. The PPG recordings showed stable heart activity over long periods of time in most animals, but in some animals the heart rates were more variable. Moreover, the population showed considerable animal-to-animal variability in resting heart rate. Interestingly, the heart rate of resting animals showed a multimodal distribution, both across the population and sometimes within a long stretch of data from a single animal. These data suggest that heart activity falls into states of higher or lower activity, which may be a consequence of variable metabolic needs of the animal during the time of recording, such as digestion, movement or excretion. Alternatively, the high- and low-activity states may reflect long-lasting circatidal (rhythms that are governed by the tide) (Chabot and Watson, 2010a) or circadian rhythms the animal experienced in the wild (Chabot and Watson, 2010b; De La Iglesia and Hsu, 2010). Unfortunately, we do not know how long a time period passed between when the animals were caught and when they arrived in the laboratory, so traces of environmental influences might have been differentially lost by variable times between capture and experiment.

Some animals displayed heart inhibitory bouts, periods of bradycardia during which the heart slowed or completely stopped. When seen in a given animal, they persisted throughout an entire experiment (Fig. 4). These inhibitory bouts are thought to have a physiological function, as they are linked to times of gill ventilation during reversal of pumping of the scaphognathite (McMahon, 1999).

### Responses to perturbation

The triphasic pyloric rhythm is robust to several global perturbations, including temperature (Soofi et al., 2014; Tang et al., 2010, 2012) and pH (Haley et al., 2018). Nonetheless, there is significant animal-to-animal variability in responses to such perturbations (Haddad and Marder, 2018; Tang et al., 2012), which is thought to be a consequence of variable underlying conductances in identified neurons across the population (Prinz et al., 2004; Schulz et al., 2006, 2007; Temporal et al., 2014). Interestingly, there are differential sensitivities of the responses of isolated STGs and cardiac ganglia to pH (Haley et al., 2018), with the cardiac ganglion being more stable to acid than the pyloric rhythm, while the pyloric rhythm was more stable to base (Haley et al., 2018).

The present study is the first, to our knowledge, to compare the *in vivo* responses of the heart and pylorus to any perturbation in the same species. We see here that the heart was more stable to high temperature than the pyloric rhythm. It is interesting that the two systems are differentially robust to perturbations.

### The origin of the coherence

Although many of the recordings showed coherence between the heart and pyloric recordings measured on the pyloric PPG, coherence was not seen in direct electrical recordings. Moreover, when the heart was silent, the pyloric PPG showed a clear single band associated with the pyloric rhythm, and there were numerous instances when the two rhythms were quite different. This indicates that the coherence is not due to cross-talk between the sensors themselves. The coherence between the two signals picked up by the pyloric PPG sensor is likely either due to it picking up pulsations of the ophthalmic artery or a consequence of a direct biomechanical coupling between the heart and the stomach, as the heart movement pulls the most posterior portion of the stomach with each heartbeat. If one opens the carapace, the pulsations of the hemolymph are visible, and it is sometimes possible to see the stomach position changing with each heartbeat. Therefore, either or both of these cases are possible, especially as the ophthalmic artery runs between the paired dorsal dilator muscles (the source of the pyloric PPG signal). It is important to reiterate that the frequencies of the two rhythms vary, and that across animals there are not fixed phases between them. This suggests that while neuromodulatory tone might influence both cardiac ganglion and the STG, there appears to be no strong, obligatory common drive.

### Physiological implications

*Cancer* *borealis* experiences wide temperature ranges in its natural habitat. Crabs can live for weeks without eating, but presumably cannot survive for extended periods of time without hemolymph oxygenation and circulation. This suggests that the fact that the critical temperature for the heart rhythm is higher than that for the pyloric rhythm may contribute to the animal's survival. The additional 4–5°C might make a large difference for an animal caught by outgoing tides in shallow water during the summer, and give it time to find its way to more hospitable environments. Interestingly, the mean critical temperatures are within the range that *C. borealis* might experience during a New England summer in shallow water or intertidal adventures. The animal-to-animal variability in these critical temperatures may be important signatures that explain the survival of some animals during the summer heat.
